# VarMod: modelling the functional effects of non-synonymous variants

**DOI:** 10.1093/nar/gku483

**Published:** 2014-06-06

**Authors:** Morena Pappalardo, Mark N. Wass

**Affiliations:** Centre for Molecular Processing, School of Biosciences, University of Kent, CT2 7NH, UK

## Abstract

Unravelling the genotype–phenotype relationship in humans remains a challenging task in genomics studies. Recent advances in sequencing technologies mean there are now thousands of sequenced human genomes, revealing millions of single nucleotide variants (SNVs). For non-synonymous SNVs present in proteins the difficulties of the problem lie in first identifying those nsSNVs that result in a functional change in the protein among the many non-functional variants and in turn linking this functional change to phenotype. Here we present VarMod (Variant Modeller) a method that utilises both protein sequence and structural features to predict nsSNVs that alter protein function. VarMod develops recent observations that functional nsSNVs are enriched at protein–protein interfaces and protein–ligand binding sites and uses these characteristics to make predictions. In benchmarking on a set of nearly 3000 nsSNVs VarMod performance is comparable to an existing state of the art method. The VarMod web server provides extensive resources to investigate the sequence and structural features associated with the predictions including visualisation of protein models and complexes via an interactive JSmol molecular viewer. VarMod is available for use at http://www.wasslab.org/varmod.

## INTRODUCTION

The ability to sequence genomes has resulted in the identification of millions of genetic variants, particularly single nucleotide variants (SNVs), within the human population as highlighted by the 1000 genomes project (1,2). Additionally, other studies have demonstrated that individuals have many rare SNVs (3,4). The data generated by such studies provide a unique resource for investigating the genotype to phenotype relationship. However, this is a complex problem as demonstrated by Genome Wide Association Studies (GWAS), which have identified many variants associated with disease risk but have only explained a limited amount of heritability (5). Additionally, in these studies, it is difficult to identify causal variants from a selection of candidate SNVs in the regions of the genome associated with the particular disease.

There is therefore a need to develop methods to identify SNVs, in our case non-synonymous SNVs (nsSNVs), that are likely to affect the function of the protein in which they are present and are more likely to be associated with a change in phenotype. A number of methods have been developed previously (reviewed in (6)), with the Sorting Intolerant From Tolerant algorithm (SIFT, 7) and PolyPhen (8) being among the most well known. SIFT uses residue conservation in multiple sequence alignments to identify function altering nsSNVs, while PolyPhen uses machine learning to combine features from both sequence and structure.

Here we have developed VarMod a new method for identifying functional nsSNVs. VarMod develops our recent research in which we demonstrated that disease associated nsSNVs are enriched at protein–protein interfaces (9). Additionally, in GWAS, we have previously used structural modelling of ligand binding sites to identify likely candidates for association with disease (10–12). For example, in a kidney disease genome wide association study (10), we demonstrated that the variant rs13538 results in a phenylalanine to serine change located in the acetyl Co-enzymeA binding site of the protein NAT8 and proposed that the variant may have an effect on the activity of the enzyme (10). VarMod builds upon these observations and uses structural modelling of ligand binding and protein–protein interface sites to generate features that are combined with other features such as residue to conservation to identify functional nsSNVs. The VarMod web server provides an overall prediction made using a machine learning approach (a support vector machine) to combine the data from the different individual analyses. Additionally the server provides users with extensive resources to investigate the results from the separate analyses.

## METHODS

### The VarMod algorithm

VarMod obtains features from multiple analyses, which are combined using a support vector machine (SVM) (13) to make an overall prediction. The data sources used are described below. Sequence conservation is calculated using Jensen–Shannon divergence (14). Homologues of the query sequence are identified by PSI-BLAST (15) using an approach shown to optimise results (16), where the query sequence is initially searched against UniRef50 to generate a sequence profile that is used to search against the full UniProt sequence database (17). The query sequence and homologues are aligned using MUSCLE (18) and the resulting multiple sequence alignment used to calculate the Jensen–Shannon divergence.

To perform the structural analysis, a structural model of the query protein is generated. To do this, template structures in the protein databank (PDB) (19) are identified using hhblits (20) by searching a PDB sequence database representative at 70% sequence identity. Templates are selected with an hhblits probability (probability that the template and query sequence are homologous) score >80% and such that as much of the sequence is covered without redundantly modelling the same region of the protein multiple times. Initial structural models are generated using an approach based on the one used by Phyre2 (21,22). Side chains are added and optimised using pulchra (23). Small molecule binding sites are modelled using 3DLigandSite (with default parameters) (24) with the structural model used as the input.

Protein–protein interface sites are modelled using an approach based on Interactome3D (25). The Interactome3D high confidence set of protein–protein interactions with template complexes in the PDB was used to generate models of the complexes. For each sequence–template pair the sequence is modelled using the template by applying the structural modelling approach described above.

The features used in the SVM fall into two areas of sequence and structural features (a full list is available in Supplementary Table S1). The sequence features include residue conservation (the Jensen–Shannon convergence) and three features that represent the change of amino acid properties of size/mass, charge and functional group. The size/mass change of the amino acid is represented by the ratio of the mass of the two amino acids. To consider the change in charge between the two amino acids, the 20 amino acids are grouped according to charge (Supplementary Table S2). The feature representing the change in the charge of the amino acid considers changes between these charge groups, with values set in Supplementary Table S3. A further feature represents the change of chemical functional group present in the amino acid side chain. The amino acids are grouped as described by Innis *et al.* (26) (Supplementary Table S4) and the feature captures changes between these functional groups.

The structural features use the ligand binding site, interface site and general structural features of the model. Where ligand-binding sites have been identified the distance of the variant to the binding site is calculated and used as a feature. When a variant is in a binding site, two further features capture results from the 3DLigandSite analysis. Where interface sites have been predicted, a further feature represents the distance of the variant to an interface site. Two features represent the type of secondary structure that the variant is located in. The first uses the secondary structure types classified by DSSP (27,28), while a second feature reduces these to the three main categories of helix, sheet and coil. A final feature represents the solvent accessibility (calculated using DSSP).

The features generated are input into each of the five optimised SVM models generated during cross-validation (details below) to predict whether each variant is functional or non-functional. The outputs from each of the SVM models are converted to probabilities as described in Platt (29). An ensemble approach is taken with the probability from each SVM model weighted according to its accuracy in cross validation. The weighted probabilities are summed and normalised to generate a final probability for the VarMod prediction.

### Generating a test set

Dataset 5 from VariBench (30) was used to train and test VarMod. This dataset contains human pathogenic and neutral variants, excludes cancer mutations and is clustered so that protein sequences share no >30% sequence identity. This set was initially split with 1401 pathogenic and 1527 neutral variants retained for final testing. The remaining 11 336 pathogenic and 12 737 neutral variants were split into five groups by protein sequence to perform 5-fold cross-validation to ensure that variants from each individual sequence appear in only 1-fold.

### SVM training

The SVMs were generated by SVMlight (31) using a linear kernel. For each of the 5-folds, three were used for training, a further fold was used for validation and the SVM tested on the remaining fold. The SVMs were optimised for the trade off between training error and margin and also the cost factor to identify how training errors on positive examples should outweigh those on negative examples.

### Comparison with PolyPhen-2

To compare VarMod performance with PolyPhen-2, the final test set of nsSNVs was run on the PolyPhen-2 web server (on 1 March 2014). Predictions were made using the two different classifiers available (HumDiv and HumVar) with default settings. The ROC and Precision–Recall analyses of PolyPhen-2 were performed by varying the ‘pph2_prob’ score.

### EVALUATING VARMOD PERFORMANCE

**Figure 1.**
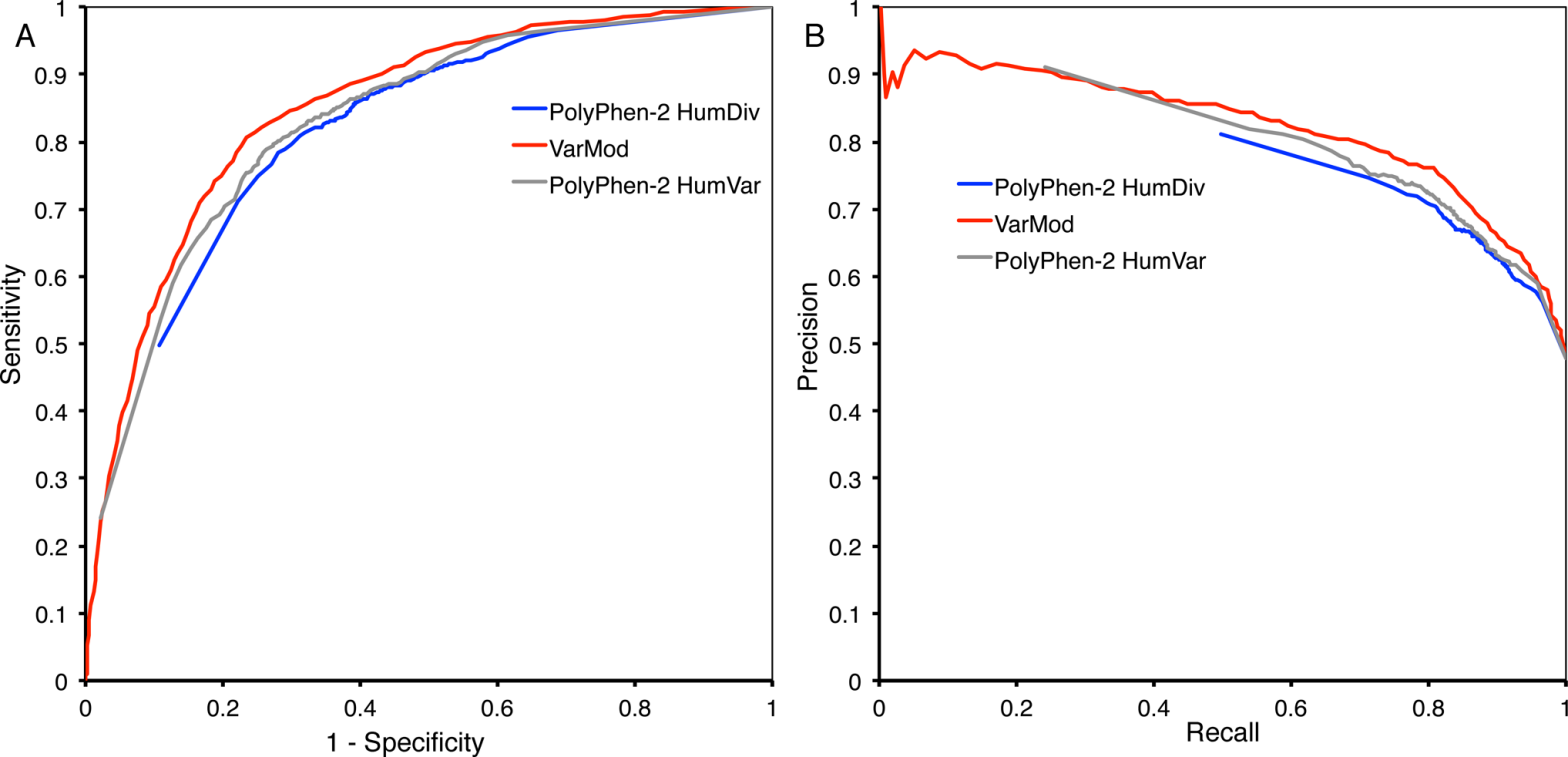
Benchmarking VarMod. Analysis of the VarMod and PolyPhen-2 predictions on the non-cross validation test set. (**A**) ROC analysis, (**B**) precision–recall graph.

The performance of VarMod was assessed using the set of sequences from VariBench that were not used in cross-validation. The performance of VarMod on the test set of sequences was assessed using the measures of specificity, sensitivity (recall), precision and a Receiver Operator Characteristic (ROC) analysis. The ROC curve and Precision–Recall graph in Figure 1 show the performance of VarMod and the comparison with PolyPhen-2. It shows that VarMod performance is comparable to PolyPhen-2. Interestingly, in the ROC analysis, neither of the PolyPhen-2 classifiers reaches the point 0,0 which is due to a small number of high confidence false positive predictions (i.e. neutral variants predicted to be pathogenic). This may reflect that PolyPhen-2 has been trained using different sets of pathogenic and neutral variants. It has also been previously observed that there is limited overlap between the predictions of different methods (32).

### The VarMod WEB SERVER

The VarMod web server is available at http://www.wasslab.org/varmod. Users are required to submit a protein sequence (raw sequence or FASTA formatted) or a UniProt accession, and a list of variant positions (e.g. A45C, where the single letter code is used to define the amino acids). A UniProt accession is required to perform the protein–protein interface analysis (optional). Processing time for each submission varies from 5 min to a few hours. Structural data has been pre-computed for all of the UniProt human principal protein isoforms, so submissions using these sequences are processed in a few minutes. Where other sequences are submitted, the structural models and binding sites need to be modelled thereby increasing the running time to a few hours.

### Results output

**Figure 2.**
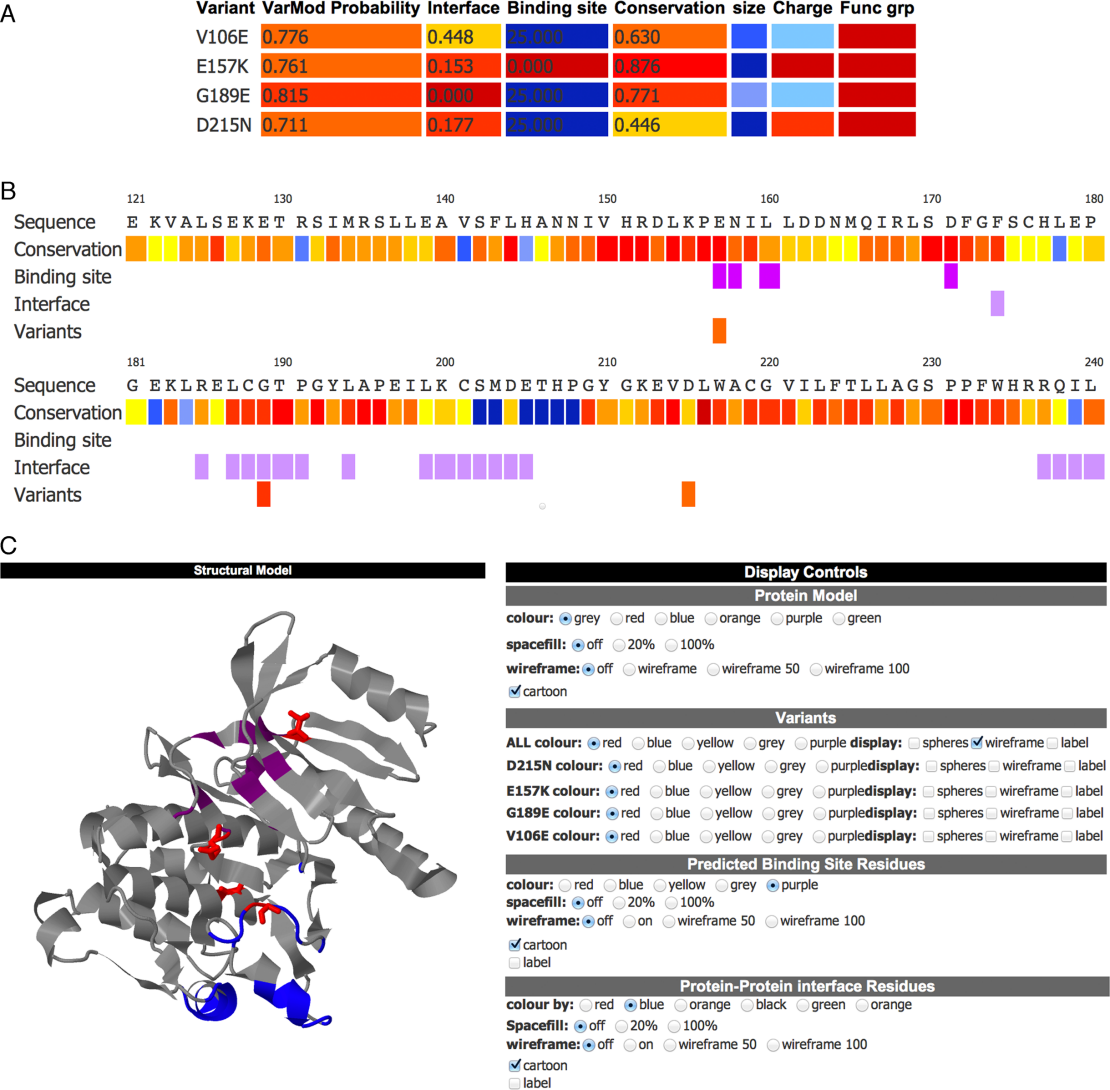
Display of VarMod results. The results for variants in Phosphorylase b kinase gamma catalytic chain (UniProt accession P15735). The variants shown are known to have a role in Glycogen storage disease 9C. (**A**) The prediction summary table, showing the overall VarMod prediction and summarising the output from the different analyses. Results are colour coded to indicate the likely relevance of the changes, with features that suggest the variant is likely to be functional coloured red with the colour scale ranging to blue for features that are least likely to lead to functional changes. (**B**) The VarMod sequence display, residues are coloured to indicate conservation and the presence of ligand binding and interface sites. (**C**) The VarMod structural view.

**Figure 3.**
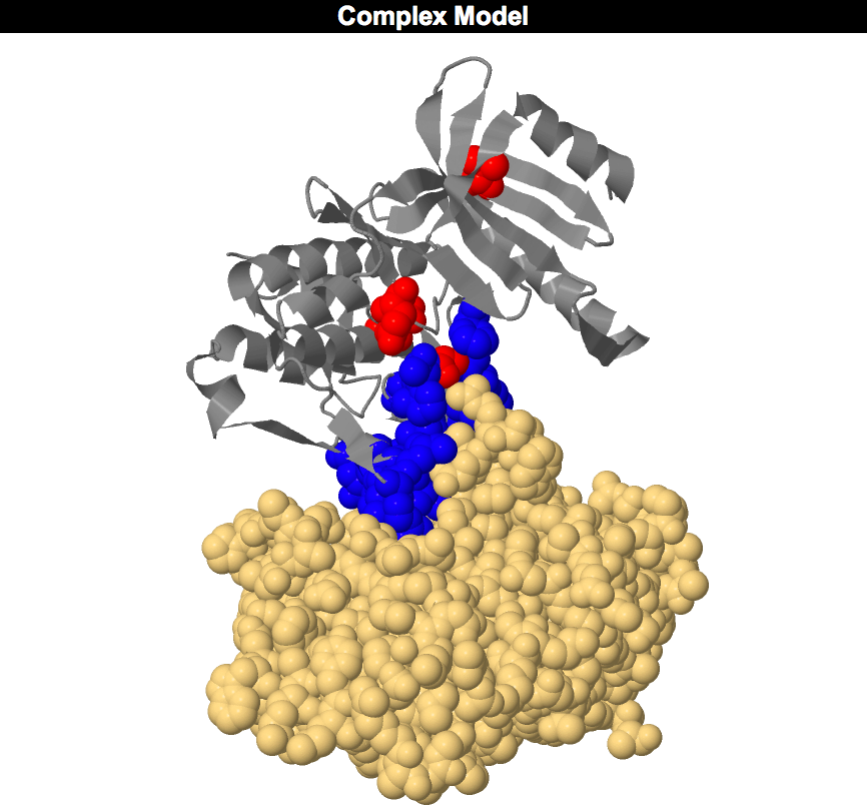
The VarMod interactions view for investigating variants located at protein–protein interfaces.

The display of VarMod results is split into multiple sections (Figures 2 and 3). The first section provides a summary table of the analyses performed and the overall prediction made for each of the submitted nsSNVs. This table is colour coded to highlight the results from the individual analyses/features to indicate if they suggest the variant could affect protein function. For example, the binding site column is coloured red if the variant is in the binding site and the colour changes to blue the more distant the variant is from a known ligand-binding site. The summary table enables the user to see the overall result and to identify analyses that may be of interest for further inspection.

The sequence and structure sections display the main analyses. The sequence section displays the protein sequence, colour coded to highlight multiple features including residue conservation, ligand binding sites and protein–protein interfaces. The summary results and sequence view can be downloaded as a PDF file.

The structural section first displays the details of the structural templates and models of the protein that have been generated (one for each region/domain for which a template was identified). A JSmol (www.jmol.org) molecular viewer forms the main part of the structural section and initially displays the model with the highest confidence (probability from hhblits alignment). The JSmol viewer enables visualisation of the modelled protein and by default is coloured to highlight the functional regions of the protein (ligand-binding and protein–protein interface sites) and the nsSNVs (red). A control panel to the right of the display enables the user to investigate the nsSNVs by displaying a different model, or modifying the display style (cartoon/spacefill or sticks representations) and colour of the whole protein, nsSNVs or functional sites. The user is able to generate high quality images of the displayed model by clicking on the ‘generate image’ button, enabling the analysis to be used for reports or publications.

The location of the nsSNVs in relation to the protein–protein interface sites can be explored further via the modelled complexes. The complex models are listed in a table, which also indicates the nsSNVs that are present in the model and if they occur within an interface. The complexes can be viewed in a separate JSmol viewer accessed from a link for each of the entries in the list.

## CONCLUDING REMARKS

VarMod was developed to use recent observations that disease associated nsSNVs are frequently located at ligand-binding and protein–protein interface sites and to automate manual approaches that we have previously used to analyse GWAS candidate nsSNVs. We have demonstrated that VarMod performance on a large and established benchmark set is comparable to an existing state of the art method (PolyPhen-2). The VarMod server provides a resource for users to identify functional nvSNVs and to investigate the individual features associated with these variants. Plans for future improvements to the server include increasing the number of interface and binding site features such as considering how variants may alter binding energies and options to submit variants in alternative formats such as Variant Call Files (VCF), which will facilitate high throughput analysis of nsSNVs identified from sequencing studies.

## SUPPLEMENTARY DATA

Supplementary Data are available at NAR Online.

Supplementary Data

## References

[1] 1000 Genomes Project Consortium (2010). A map of human genome variation from population-scale sequencing. Nature.

[2] Abecasis G.R., Auton A., Brooks L.D., DePristo M.A., Durbin R.M., Handsaker R.E., Kang H.M., Marth G.T., McVean G.A., 1000 Genomes Project Consortium (2012). An integrated map of genetic variation from 1092 human genomes. Nature.

[3] Nelson M.R., Wegmann D., Ehm M.G., Kessner D., St Jean P., Verzilli C., Shen J., Tang Z., Bacanu S.-A., Fraser D. (2012). An abundance of rare functional variants in 202 drug target genes sequenced in 14 002 people. Science.

[4] Tennessen J.A., Bigham A.W., O'Connor T.D., Fu W., Kenny E.E., Gravel S., McGee S., Do R., Liu X., Jun G. (2012). Evolution and functional impact of rare coding variation from deep sequencing of human exomes. Science.

[5] Eichler E.E., Flint J., Gibson G., Kong A., Leal S.M., Moore J.H., Nadeau J.H. (2010). Missing heritability and strategies for finding the underlying causes of complex disease. Nat. Rev. Genet..

[6] Peterson T.A., Doughty E., Kann M.G. (2013). Towards precision medicine: advances in computational approaches for the analysis of human variants. J. Mol. Biol..

[7] Sim N.L., Kumar P., Hu J., Henikoff S., Schneider G., Ng P.C. (2012). SIFT web server: predicting effects of amino acid substitutions on proteins. Nucleic Acids Res..

[8] Adzhubei I.A., Schmidt S., Peshkin L., Ramensky V.E., Gerasimova A., Bork P., Kondrashov A.S., Sunyaev S.R. (2010). A method and server for predicting damaging missense mutations. Nat. Methods.

[9] David A., Razali R., Wass M.N., Sternberg M.J.E. (2012). Protein-protein interaction sites are hot spots for disease-associated nonsynonymous SNPs. Hum. Mutat..

[10] Chambers J.C., Zhang W., Lord G.M., Van der Harst P., Lawlor D.A., Sehmi J.S., Gale D.P., Wass M.N., Ahmadi K.R., Bakker S.J.L. (2010). Genetic loci influencing kidney function and chronic kidney disease. Nat. Genet..

[11] Chambers J.C., Zhang W., Sehmi J., Li X., Wass M.N., Van der Harst P., Holm H., Sanna S., Kavousi M., Baumeister S.E. (2011). Genome-wide association study identifies loci influencing concentrations of liver enzymes in plasma. Nat. Genet..

[12] Chambers J.C., Zhang W., Li Y., Sehmi J., Wass M.N., Zabaneh D., Hoggart C., Bayele H., McCarthy M.I., Peltonen L. (2009). Genome-wide association study identifies variants in TMPRSS6 associated with hemoglobin levels. Nat. Genet..

[13] Vapnik V.N. (1999). An overview of statistical learning theory. IEEE Trans. Neural Netw..

[14] Capra J M., Singh M. (2008). Characterization and prediction of residues determining protein functional. Bioinformatics.

[15] Altschul S.F., Madden T.L., Schaffer A.A., Zhang J., Zhang Z., Miller W., Lipman D.J. (1997). Gapped BLAST and PSI-BLAST: a new generation of protein database search programs. Nucleic Acids Res..

[16] Chubb D., Jefferys B.R., Sternberg M.J., Kelley L.A. (2010). Sequencing delivers diminishing returns for homology detection: implications for mapping the protein universe. Bioinformatics.

[17] UniProt Consortium (2012). Reorganizing the protein space at the Universal Protein Resource (UniProt). Nucleic Acids Res..

[18] Edgar R.C. (2004). MUSCLE: multiple sequence alignment with high accuracy and high throughput. Nucleic Acids Res..

[19] Rose P.W., Bi C., Bluhm W.F., Christie C.H., Dimitropoulos D., Dutta S., Green R.K., Goodsell D.S., Prlic A., Quesada M. (2013). The RCSB Protein Data Bank: new resources for research and education. Nucleic Acids Res..

[20] Remmert M., Biegert A., Hauser A., Söding J. (2012). HHblits: lightning-fast iterative protein sequence searching by HMM-HMM alignment. Nat. Methods.

[21] Kelley L.A., Sternberg M.J. (2009). Protein structure prediction on the web: a case study using the Phyre server. Nat. Protoc..

[22] Bennett-Lovsey R.M., Herbert A.D., Sternberg M.J., Kelley L.A. (2008). Exploring the extremes of sequence/structure space with ensemble fold recognition in the program Phyre. *Proteins*.

[23] Rotkiewicz P., Skolnick J. (2008). Fast procedure for reconstruction of full-atom protein models from reduced representations. J. Comput. Chem..

[24] Wass M.N., Kelley L.A., Sternberg M.J.E. (2010). 3DLigandSite: predicting ligand-binding sites using similar structures. Nucleic Acids Res..

[25] Mosca R., Ceol A., Aloy P. (2012). Interactome3D: adding structural details to protein networks. Nat. Methods.

[26] Innis C.A., Anand A.P., Sowdhamini R. (2004). Prediction of functional sites in proteins using conserved functional group analysis. J. Mol. Biol..

[27] Joosten R.P., Beek te, T.A.H., Krieger E., Hekkelman M.L., Hooft R.W.W., Schneider R., Sander C., Vriend G. (2011). A series of PDB related databases for everyday needs. Nucleic Acids Res..

[28] Kabsch W., Sander C. (1983). Dictionary of protein secondary structure: pattern recognition of hydrogen-bonded and geometrical features. Biopolymers.

[29] Platt J. (1999). Probabilistic outputs for support vector machines and comparisons to regularized likelihood methods.

[30] Sasidharan Nair P., Vihinen M. (2013). VariBench: a benchmark database for variations. Hum. Mutat..

[31] Joachims T., Schölkopf B, Burges C, Smola A (1999). Making large-scale SVM learning practical. *Advances in Kernel Methods—Support Vector Learning*.

[32] Chun S., Fay J.C. (2009). Identification of deleterious mutations within three human genomes. Genome Res..

