# Supplementary material for: VarMod: modelling the functional effects of non-synonymous variants
Source: Nucleic Acids Res. 2014 Jun 6;42(Web Server issue):W331–6. doi: 10.1093/nar/gku483 (PMC4086131; doi:10.1093/nar/gku483)
Supplement: Supplementary Data [file supp_42_W1_W331__index.html]

Supplementary Data 

# VarMod: modelling the functional effects of non-synonymous variants

## Supplementary Data

**Files in this Data Supplement:**

- SUPPLEMENTARY DATA
