# Supplementary material for: VarMod: modelling the functional effects of non-synonymous variants
Source: Nucleic Acids Res. 2014 Jun 6;42(Web Server issue):W331–6. doi: 10.1093/nar/gku483 (PMC4086131; doi:10.1093/nar/gku483)
Supplement: Supplementary Data [file supp_gku483_nar-00630-web-b-2014-File005.pdf]

VarMod: Modelling the functional effects of non-synonymous variants

Morena Pappalardo & Mark N. Wass*

Centre for Molecular Processing, School of Biosciences, University of Kent, UK.

* To whom correspondence should be addressed: m.n.wass@kent.ac.uk

Supplementary methods and Tables

The text and tables below explain the groupings used for the different amino acid properties and how they were converted to features for input into the support vector machine (SVM). Supplementary table 1 displays the full list of features input into the SVM. The weight of each of the features used in the SVM was calculated using the script provided with SVMlight, which calculates the weighted sum of the support vectors. It shows that the Jensen Shannon conservation score has the highest weighted followed by the binding site and interface site features and solvent accessibility features. Conservation (Jensen Shannon divergence) has been used previously by other methods including SIFT and PolyPhen and it is not surprising that it is weighted highly. The weighting of the interface and binding site features demonstrates that they used by VarMod to make predictions and are more informative than other features such as those relating to secondary structure.

Feature	Value range	SVM weight
js convergence score (conservation)	0-1	1.93
Amino acid properties		
amino acid charge change	see supplementary table 2	0.35
amino acid mass change	See supplementary table 3	0.29
amino acid functional group change	1 where functional change, 0 otherwise (see Supplementary table 4)	0.41
3DLigandSite features		
distance to binding site	0-1 (actual distance divided by 25, values greater than 1 are rounded down to 1)	1.09
3DLigandSite average distance to ligands	0-1 (value/ 2)	1.51
3DLigandSite number of ligands that bind to this residue	num/50	0.80
Interface site features		
distance to interface site	0-1 (distance/25, values greater than 1 round down to 1)	1.18
Secondary Structure features		
DSSP -secondary structure- B	0/1 (1 if ss is B, 0 otherwise)	0.47
DSSP -secondary structure- G	0/1 (1 if ss is G, 0 otherwise)	0.09
DSSP -secondary structure- I	0/1 (1 if ss is I, 0 otherwise)	0.26
DSSP -secondary structure- T	0/1 (1 if ss is T, 0 otherwise)	0.13
DSSP -secondary structure- S	0/1 (1 if ss is S, 0 otherwise)	0.11
DSSP -secondary structure- BL	0/1 (1 if ss is BL, 0 otherwise)	0.20
DSSP -secondary structure- H	0/1 (1 if ss is H, 0 otherwise)	0.13
DSSP -secondary structure-E	0/1 (1 if ss is E, 0 otherwise)	0.02
		0.48
DSSP -secondary structure Type - Heilx	0/1 (1 if ss type is is H, 0 otherwise)	0.49
DSSP -secondary structure Type – Strand	0/1 (1 if ss is B, 0 otherwise)	0.45
DSSP -secondary structure Type - Coil	0/1 (1 if ss is B, 0 otherwise)	0.08
distance from end of secondary structure	0 - 0.5 (0.5 in the middle, 0 at end of secondary structure element)	0.26
DSSP - solvent accessibility	0-1 (solvent accessibility / 300)	-1.05

Supplementary Table 1. The SVM features used in VarMod are listed with the value range used for each feature and the weighting of the features in the SVM.

Supplementary Tables 2-4 relate to the change in amino acid properties of the variants. Supplementary Table 2 shows the amino acid charge groups and Supplementary table 3 shows the value for the amino acid charge feature for changes between these groups. Supplementary table 4 shows the groups of amino acids based on functional groups present in the side chain. The feature associated with functional groups is either 0 (no change in functional group), 1 (change in functional group).

**Charge group**	**Amino acids**
Positive charge	R, H, K
Negative charge	D, E
Negative polar	N, Q
Positive polar	S, T
Hydrophobic	G, A, V, I, L, M, F, Y, W, C, P

Supplementary Table 2. Amino acid charge groups.

	Positive charge	Negative charge	Negative polar	Positive polar	Hydrophobic
Positive charge	0				
Negative charge	1	0			
Negative polar	0.5	0.25	0		
Positive polar	0.25	0.5	0.75	0	
Hydrophobic	1	1	0.75	0.75	0

Supplementary Table 3. SVM feature value for change in amino acid charge.

**Functional group**	**Amino acids**
Positive	R, H, K
Carboxylate	D, E
Phenyl	F, Y, W
hydroxyl	S, T, Y
Amido	N, Q
Other/none	G, A, V, I, L, M, C, P

Supplementary Table 4. Amino acid functional groups used as defined in Innis et al., (28).
